# Predicting Bone Metastasis Risk Based on Skull Base Invasion in Locally Advanced Nasopharyngeal Carcinoma

**DOI:** 10.3389/fonc.2022.812358

**Published:** 2022-04-07

**Authors:** Bo Wu, Yu Guo, Hai-hua Yang, Qian-gang Gao, Ye Tian

**Affiliations:** ^1^ Department of Radiotherapy and Oncology, The Second Affiliated Hospital of Soochow University, Suzhou, China; ^2^ Department of Radiotherapy, Taizhou Central Hospital (Taizhou University Hospital), Taizhou, China; ^3^ Department of Radiotherapy, Taizhou Hospital, Linhai, China

**Keywords:** nasopharyngeal carcinoma, skull base invasion, bone metastasis, bone metastasis-free survival, prediction model, nomogram, intensity modulated radiation therapy

## Abstract

**Objective:**

To develop and validate a bone metastasis prediction model based on skull base invasion (SBI) in patients with locally advanced nasopharyngeal carcinoma (LA-NPC).

**Methods:**

This retrospective cohort study enrolled 290 patients with LA-NPC who received intensity-modulated radiation therapy in two hospitals from 2010 to 2020. Patient characteristics were grouped by SBI and hospital. Both unadjusted and multivariate-adjusted models were used to determine bone metastasis risk based on SBI status. Subgroup analysis was performed to investigate heterogeneity using a forest graph. Cox proportional hazard regression analysis was used to screen for risk factors of bone metastasis-free survival (BMFS). A nomogram of BMFS based on SBI was developed and validated using C-index, receiver operating characteristic curve, calibration curves, and decision curve analysis after Cox proportional hazard regression analysis.

**Results:**

The incidence of bone metastasis was 14.83% (43/290), 20.69% (24/116), and 10.92% (19/174) in the overall population, SBI-positive group, and SBI-negative group, respectively. In the unadjusted model, SBI was associated with reduced BMFS [HR 2.43 (1.32–4.47), *P* = 0.004], and the results remained stable after three continuous adjustments (*P <*0.05). No significant interaction was found in the subgroup analyses (*P* for interaction >0.05). According to Cox proportional hazard regression analysis and clinical value results, potential risk factors included SBI, Karnofsky performance status, TNM stage, induction chemotherapy, concurrent chemoradiotherapy, and adjuvant chemotherapy. Using a training C-index of 0.80 and a validation C-index of 0.79, the nomogram predicted BMFS and demonstrated satisfactory prognostic capability in 2, 3, and 5 years (area under curve: 83.7% vs. 79.6%, 81.7% vs. 88.2%, and 79.0% vs. 93.8%, respectively).

**Conclusion:**

Skull base invasion is a risk factor for bone metastasis in patients with LA-NPC. The SBI-based nomogram model can be used to predict bone metastasis and may assist in identifying LA-NPC patients at the highest risk of bone metastasis.

## Introduction

Nasopharyngeal carcinoma (NPC), a squamous cell carcinoma that develops on the nasopharyngeal epithelium, is one of the most common malignant tumors in South China, with more than 70% of patients diagnosed with locally advanced NPC (LA-NPC) (1–3). Although treatments like intensity-modulated radiation therapy (IMRT) can improve local control rate, the incidence of distant metastasis ranges from 11.00 to 27.08% and remains a significant concern (4–7). Multiple studies have correlated distant metastasis with poor prognosis (8, 9). NPC is associated with pulmonary, liver, and bone metastasis, with bone being the most common, occurring concurrent with or before other distant metastases (10, 11). Thus, it is critical to identify risk factors that may influence bone metastasis in LA-NPC patients.

The skull base is a common site of tumor invasion in LA-NPC patients (12). Zou et al. (13) studied 518 LA-NPC patients and found that those with skull base invasion (SBI) had a higher risk of bone metastasis than those without (16.4% vs. 6.6%, respectively; *P* < 0.05). Other studies have shown that SBI detected by computed tomography (CT) was predictive of bone metastasis in patients with early N-stage NPC [2.478 (1.146–5.358), *P* = 0.021] (14). However, more research is needed to determine the independent prognostic value of SBI to the risk of bone metastasis. Furthermore, there is no international consensus on the best model to predict bone metastasis in LA-NPC patients based on SBI (15).

A nomogram is a visual depiction of a complicated mathematical formula that offers the overall likelihood of a specific outcome (16). Nomograms generated by regression analysis are widely used in regimen selection, tumor recurrence/metastasis prediction, and prognostic evaluation (17, 18). In addition, the prediction model can be integrated into TNM staging (19). This retrospective study was designed to assess the relationship between SBI and bone metastasis and to develop a bone metastasis risk model based on SBI.

## Materials and Methods

### Study Design and Data Source

**Figure 1:**
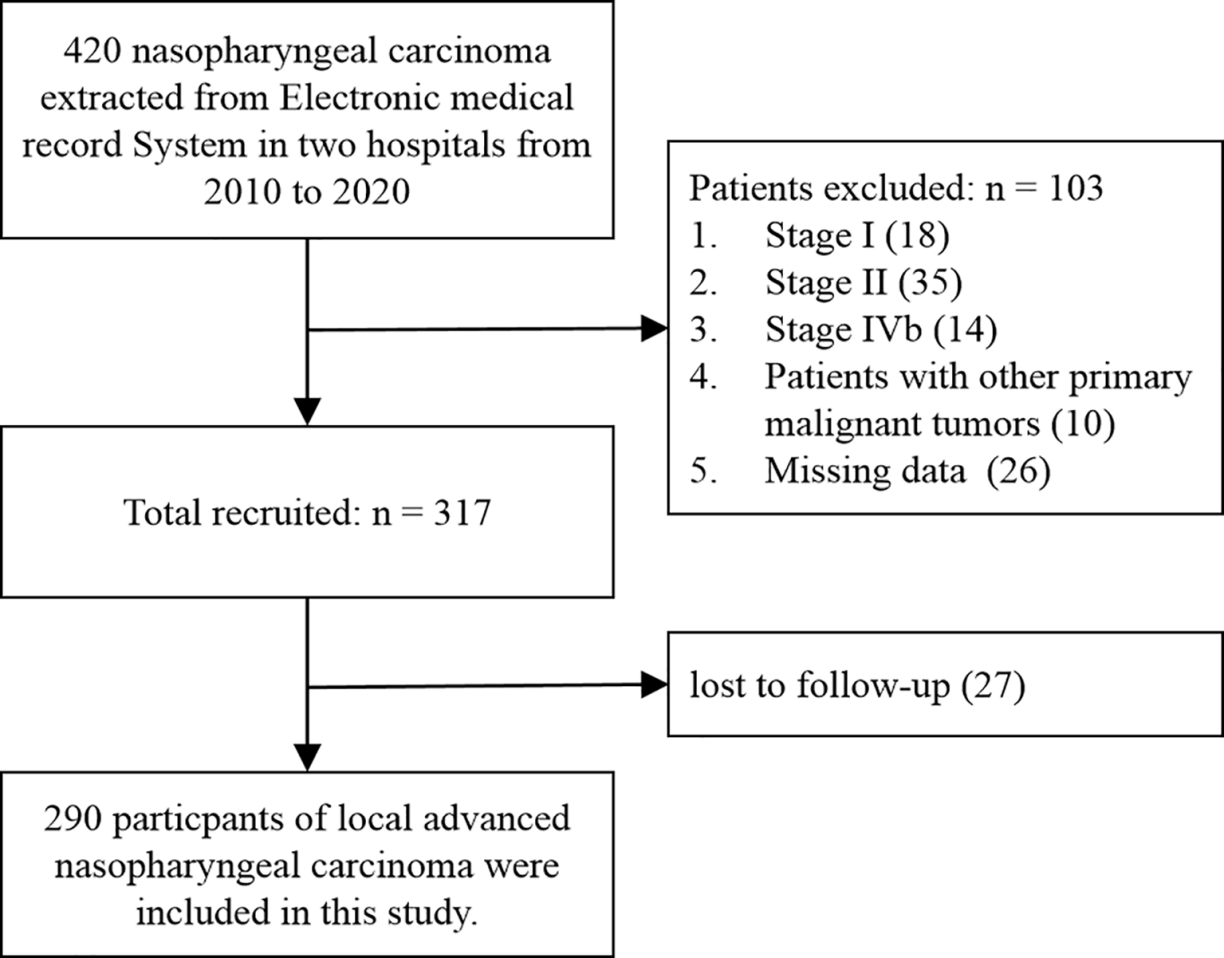
Flow diagram of the study selection process.

A retrospective study was conducted by consecutively enrolling LA-NPC patients seen at Taizhou Central Hospital (Taizhou University Hospital) and Taizhou Hospital from 2010 to 2020 (**Figure 1**). The local Institutional Review Boards approved the study (No. 2019-SC-019, Date: 2019/06/09). Because the study was retrospective, the requirement for informed consent was waived.

Inclusion criteria included (1) a pathological diagnosis of NPC, (2) complete imaging results confirming LA-NPC (stage III or IVa, AJCC 8th edition), (3) CT or magnetic resonance imaging (MRI) diagnosis of SBI, and (4) receipt of IMRT alone or in combination with induction chemotherapy (IC), concurrent chemoradiotherapy (CCRT), or adjuvant chemotherapy (AC). Exclusion criteria included (1) stage I, II, and IVb (n = 67), (2) presence of other primary malignant tumors (n = 10), (3) incomplete clinical data (n = 26), and (4) loss to follow-up (n = 27). Based on these criteria, 290 LA-NPC patients were included in the study.

The times from inclusion in the study to bone metastasis, distant metastasis, or death were defined as bone metastasis-free survival (BMFS), distant metastasis-free survival (DMFS), or overall survival (OS), respectively. Follow-up was conducted during outpatient visits or by phone every 3 months for the first 2 years and every 6 months for the next 3–5 years. The end of follow-up was defined as the date of death or June 2021.

### Predictor Variables

Potential predictor variables were collected before and during treatment. Patient information, including demographics, clinical features, imaging findings, and treatment, was obtained from the hospital information systems. SBI was separately assessed for each patient by two radiologists using contrast-enhanced CT and/or MRI (14, 20). Any disagreements were reviewed until a consensus was reached.

All enrolled LA-NPC patients were treated with IMRT as described previously (21, 22). In brief, the prescription doses of 70-76Gy, 66-70Gy, 60-66Gy, or 56-60Gy were administered to the primary tumor volume of the gross tumor (GTVnx) and the involved lymph nodes (GTVnd), with the clinical target volume including high- and low-risk regions (CTV1/2). IC, CCRT, or AC was usually recommended for these patients in the form of single-agent cisplatin or platinum-based regimen. The combination chemotherapy regimens included platinum/fluorouracil, gemcitabine/platinum, docetaxel/platinum, and docetaxel/platinum/fluorouracil (**Supplementary S1**).

### Association Analyses of Skull Base Invasion With Covariables and Outcomes

Unadjusted and multivariable-adjusted models were used to determine the relationship between SBI and LA-NPC outcomes. Covariables were added to a Cox regression model and dropped one by one. The coefficients of the corresponding regression were compared. Effect modification based on TNM stage, IC, CCRT, and AC treatment was investigated by including an interaction term with SBI in the Cox regression model for bone metastasis. The findings were presented as a hazard ratio (HR) with 95% confidence intervals (CI).

### Feature Extraction and Model Building

Univariate and multivariate Cox regression analyses were performed to identify clinically important variables related to bone metastasis risk (*P* < 0.1). A nomogram predicting bone metastasis was then established to visualize model efficiency using a training dataset from Taizhou Central Hospital (Taizhou University Hospital). The results were validated with a validation dataset from Taizhou Hospital. The area under the curve (AUC) of the receiver operating characteristic curve (ROC) was used to evaluate the accuracy of the nomogram model. The concordance index (C-index) was calculated to assess the model’s discrimination ability and a calibration curve was plotted to calibrate the model (23). The clinical usefulness of the nomogram was estimated using decision curve analysis (24).

### Statistical Analyses

Descriptive analysis was used to characterize the study participants. Categorical variables were expressed as proportions (%), and continuous variables were expressed as the mean plus standard deviation. The correlation between clinical covariables and SBI or hospital was analyzed using χ^2^ and *t* tests. *P <*0.05 denoted a statistically significant difference (two-tailed test). Pearson’s coefficients of association were calculated to assess the collinearity between SBI and the covariables. The threshold was set at r <0.6 with *P <*0.05. All data were processed using Free Statistics software version 1.3 and SPSS software version 25.0.

## Results

### Study Population

In total, 290 cases were included in the study with a median 49.5-month follow-up (range: 6–60 months). Of these, 198 cases were from Taizhou central hospital (Taizhou university hospital) and 92 cases were from Taizhou Hospital. Baseline characteristics of the patients, grouped by the presence or absence of SBI, are shown in **Table 1**. The patients were an average of 54.9 ± 11.6 years of age and 74.5% (216/290) were male. Most participants (71.3%, 207/290) had TNM stage III, while the remaining 83 had TNM stage IVa. Forty percent (116/290) of the patients had SBI. There were significant differences in T category, N category, TNM stage, and IC between the SBI-positive and SBI-negative groups (*P <*0.05). However, no statistically significant differences in hospital, age, sex, Karnofsky performance status (KPS), smoking index, histological type, CCRT, and AC were observed between the groups (*P >*0.05). **Table S1** summarizes the baseline characteristics by hospital. While there was a significant difference in CCRT (77.3% vs. 65.2%, *P* = 0.043), no statistically significant differences were reported in SBI, bone metastasis, age, sex, KPS, smoking index, histological type, T category, N category, TNM stage, IC, and AC (*P >*0.05).

**Table 1:** Baseline characteristics of 290 locally advanced nasopharyngeal carcinoma patients grouped by presence of skull base invasion.

Variable	Total (n = 290)	SBI: No (n = 174)	SBI: Yes (n = 116)	*p* value
Hospital				0.938
TZCH	198 (68.3)	118 (67.8)	80 (69)	
TZH	92 (31.7)	56 (32.2)	36 (31)	
Age(years), Mean ± SD	54.9 ± 11.6	54.7 ± 11.6	55.2 ± 11.5	0.694
Age(years)				0.981
≤55	144 (49.7)	87 (50)	57 (49.1)	
>55	146 (50.3)	87 (50)	59 (50.9)	
Sex				0.978
Female	74 (25.5)	45 (25.9)	29 (25)	
Male	216 (74.5)	129 (74.1)	87 (75)	
KPS scores				0.145
≤70	66 (22.8)	34 (19.5)	32 (27.6)	
>70	224 (77.2)	140 (80.5)	84 (72.4)	
Smoking index				1.000
≤400	207 (71.4)	124 (71.3)	83 (71.6)	
>400	83 (28.6)	50 (28.7)	33 (28.4)	
Histological type				0.751
Keratinizing	22 (7.6)	12 (6.9)	10 (8.6)	
Non-keratinizing	268 (92.4)	162 (93.1)	106 (91.4)	
T category				< 0.001
T1-2	114 (39.3)	114 (65.5)	0 (0)	
T3-4	176 (60.7)	60 (34.5)	116 (100)	
N category				0.003
N0-1	57 (19.7)	24 (13.8)	33 (28.4)	
N2-3	233 (80.3)	150 (86.2)	83 (71.6)	
TNM stage				< 0.001
III	207 (71.4)	143 (82.2)	64 (55.2)	
IVa	83 (28.6)	31 (17.8)	52 (44.8)	
IC				< 0.001
No	125 (43.1)	90 (51.7)	35 (30.2)	
Yes	165 (56.9)	84 (48.3)	81 (69.8)	
CCRT				0.849
No	77 (26.6)	45 (25.9)	32 (27.6)	
Yes	213 (73.4)	129 (74.1)	84 (72.4)	
AC				0.747
No	183 (63.1)	108 (62.1)	75 (64.7)	
Yes	107 (36.9)	66 (37.9)	41 (35.3)	

SBI, skull base invasion; TZCH, Taizhou Central Hospital (Taizhou University Hospital); TZH, Taizhou Hospital; KPS, karnofsky performance status; IC, induction chemotherapy; CCRT, concurrent chemoradiotherapy; AC, adjuvant chemotherapy.

### Association of Skull Base Invasion With Covariables and Outcomes

The incidence of bone metastasis was 14.83% (43/290), 20.69% (24/116), and 10.92% (19/174) in the study population, SBI-positive group, and SBI-negative group, respectively. Collinearity analysis revealed strong collinearity between SBI and T category (r = 0.657) (**Table S2**), while SBI and TNM stage (r = 0.293) did not show collinearity. Thus, TNM stage was chosen for subsequent analyses.

**Figure 2:**
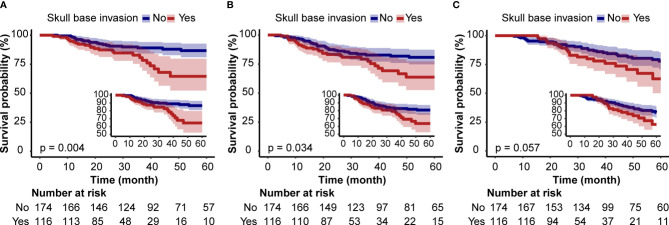
Kaplan–Meier Survival Curves for bone metastasis-free survival **(A)**, distant metastasis-free survival, **(B)** and overall survival **(C)** of locally advanced nasopharyngeal carcinoma patients based on skull base invasion.

The HRs and 95% CIs for tumor progression and survival analyses determined by SBI are shown in **Table 2**. SBI-positive patients had a shorter BMFS in the unadjusted model [HR: 2.43, 95%CI (1.32–4.47)] (**Table 2** and **Figure 2A**). After adjusting for hospital, age, sex, KPS, smoking index, histological type, TNM stage, IC, CCRT, and AC, the HRs were 2.52 (1.36–4.66), 2.28 (1.23–4.22), and 2.31 (1.17–4.54), respectively (*P <*0.05). A correlation was observed between SBI and bone metastasis in unadjusted and multivariable-adjusted models. While the Kaplan–Meier survival curve showed that SBI-positive patients had a lower DMFS and OS than SBI-negative ones, the HRs was 1.56 (0.92–2.65) and 1.56 (0.85–2.89) for DMFS and OS after adjusting for all covariables (**Table 2** and **Figures 2B, C**).

**Table 2:** Tumor progression and survival analyses by the presence of skull base invasion.

Variable	SBI	Unadjusted model		Adjusted 1^a^		Adjusted 2^b^		Adjusted 3^c^	
		HR (95%CI)	*P* value	HR (95%CI)	*P* value	HR (95%CI)	*P* value	HR (95%CI)	*P* value
BMFS	–	1		1		1		1	
	+	2.43 (1.32-4.47)	0.004	2.52 (1.36-4.66)	0.003	2.28 (1.23-4.22)	0.009	2.31 (1.17-4.54)	0.015
DMFS	–	1		1		1		1	
	+	1.75 (1.05-2.93)	0.032	1.75 (1.04-2.93)	0.034	1.69 (1.01-2.83)	0.047	1.56 (0.92-2.65)	0.098
OS	–	1		1		1		1	
	+	1.69 (0.98-2.9)	0.057	1.84 (1.06-3.21)	0.031	1.64 (0.93-2.87)	0.086	1.56 (0.85-2.89)	0.153

SBI, skull base invasion; BMFS, bone metastasis free survival; DMFS, distant metastasis free survival; OS, overall survival.

a Adjusted for hospital, age and sex.

b Adjusted for hospital, age, sex, karnofsky performance status, smoking index and histological type.

c Adjusted for all the variables.

**Figure 3:**
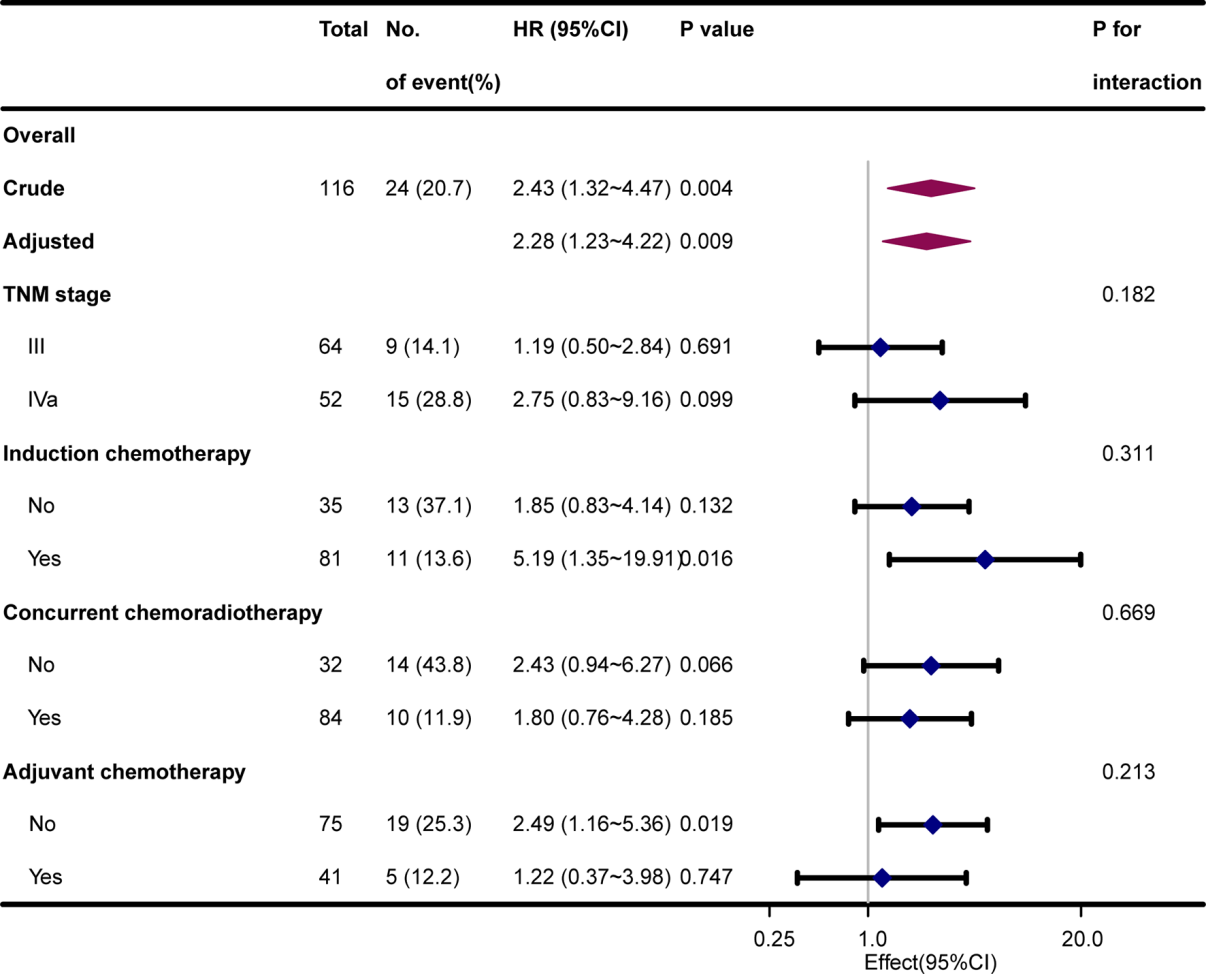
Hazard risk of bone metastasis in subgroup analyses after adjustment for hospital, age, sex, Karnofsky performance status, smoking index, and histological type.

Stratified and interactive analyses were used to determine if the relationships between SBI and bone metastasis were stable in the TNM stage, IC, CCRT, and AC subgroups (**Figure 3**). However, no significant interaction effects were found in the four subgroups (*P* for interaction > 0.05).

### Feature Selection and Model Building

**Figure 4:**
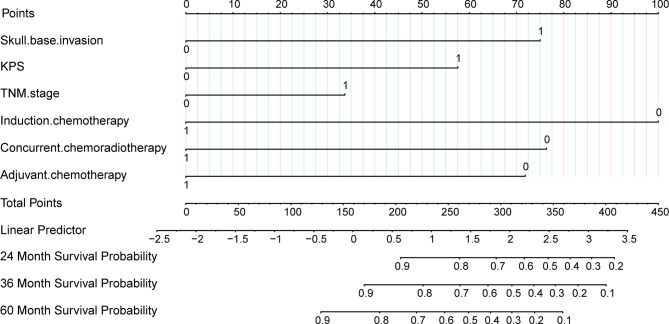
Nomogram predicting 24, 36, and 60 months of bone metastasis-free survival.

**Figure 5:**
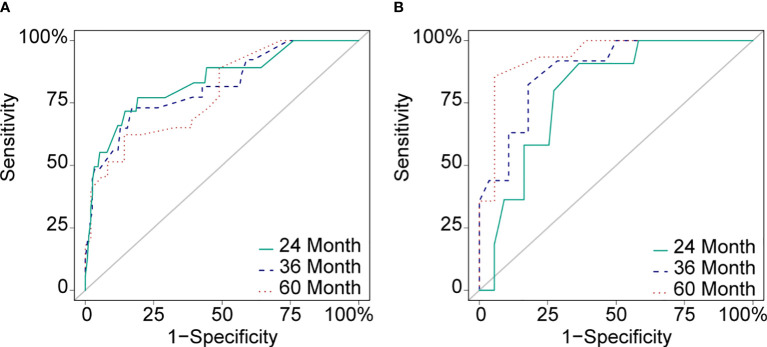
ROC curves of the training dataset **(A)** and the validation dataset **(B)** in 24 months (AUC: 83.7% vs. 79.6%), 36 months (AUC: 81.7% vs. 88.2%) and 60 months (AUC: 79.0% vs. 93.8%).

**Figure 6:**
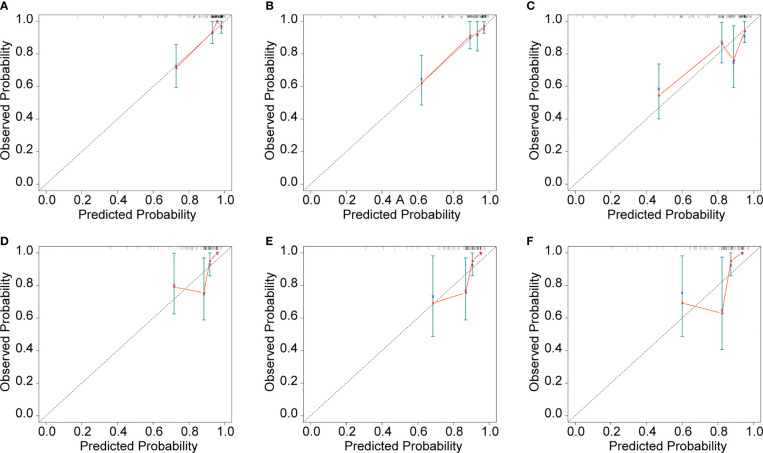
Calibration curves of the training dataset **(A–C)** and the validation dataset **(D–F)** in 24, 36, and 60 months.

**Figure 7:**
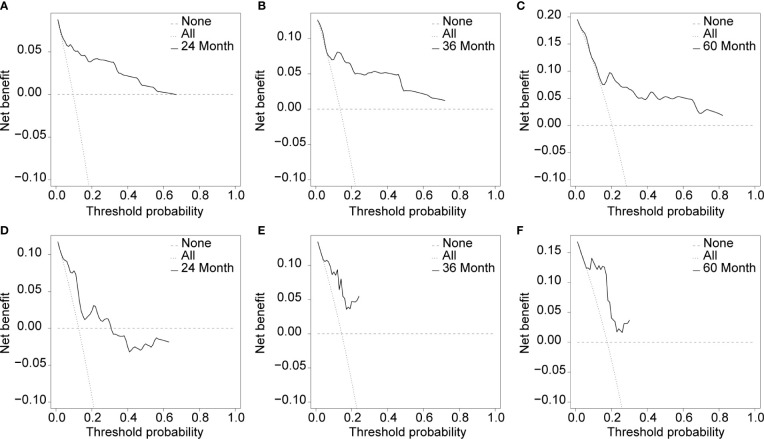
Usefulness evaluation of the training dataset **(A–C)** and the validation dataset **(D–F)** in 24, 36, and 60 months.

Cox proportional hazard regression revealed that SBI, KPS, TNM stage, IC, and CCRT were independent risk factors for BMFS (**Table 3**). AC was also selected due to its clinical value for tumor prognosis. A nomogram with the six factors is shown in **Figure 4**. The C-index for BMFS prediction in the training and validation datasets was 0.80 (95% CI 0.694–0.905) and 0.79 (95% CI 0.621–0.963), respectively. According to ROC analyses on both the training and validation datasets, the AUCs were 83.7% vs. 79.6%, 81.7% vs. 88.2%, and 79.0% vs. 93.8% for predicting 2-, 3-, and 5-year BMFS, respectively (**Figures 5A, B**). In addition, the calibration plot of the nomogram for the probability of BMFS at 2, 3, and 5 years showed strong agreement (**Figures 6A**–**F**), and the decision curve results indicated that the nomogram was clinically applicable (**Figures 7A**–**F**).

**Table 3:** Risk factors selected by Cox proportional hazard regression analysis.

Variable	Univariable		Multivariable	
	HR (95%CI)	*P* value	HR (95%CI)	*P* value
SBI: +	2.43 (1.32-4.47)	0.003	2.17 (1.13-4.15)	0.020
Age: >55	2.66 (1.39-5.11)	0.002	1.01 (0.98-1.04)	0.416
Sex: male	1.25 (0.60-2.61)	0.540		
KPS: >70	2.30 (1.24-4.27)	0.012	1.78 (0.94-3.38)	0.078
Smoking index: >400	1.53 (0.82-2.84)	0.187		
Histological type: Non-keratinizing	1.75 (0.42-7.24)	0.399		
T category: T3-4	0.83 (0.45-1.53)	0.555		
N category: N2-3	3.66 (1.13-11.83)	0.009		
TNM stage: IVa stage	2.17 (1.18-3.96)	0.014	1.84 (0.98-3.46)	0.059
IC: Yes	0.36 (0.19-0.68)	< 0.001	0.26 (0.13-0.50)	0.000
CCRT: Yes	0.26 (0.14-0.47)	< 0.001	0.31 (0.16-0.60)	0.001
AC: Yes	0.58 (0.30-1.13)	0.100	0.61 (0.30-1.27)	0.187

SBI, skull base invasion; KPS, karnofsky performance status; IC, induction chemotherapy; CCRT, concurrent chemoradiotherapy; AC, adjuvant chemotherapy.

## Discussion

Novel treatments like IMRT have steadily reduced the rate of local/regional recurrence during LA-NPC, but distant metastasis still results in treatment failures (2). According to the “seed and soil” theory, bone metastasis most often results from nutrient-rich bone tissue, chemokine and cytokine mediation, and the unique ecological niche of the bone metastasis (5, 25). The present study developed a risk prediction model by investigating the relationship between SBI and bone metastasis. SBI was significantly correlated with a higher incidence of bone metastasis and shorter BMFS. Even in multivariable-adjusted models, the results remained robust and stable. A nomogram of BMFS was developed and validated based on SBI and found to perform well in terms of calibration and discrimination.

Prior studies have assessed the risk factors for bone metastasis in NPC patients. Zhao et al. (26) suggested that bone metastasis is related to N but not T classification. Another study yielded comparable results (27). These studies did not specifically investigate the risk of bone metastasis caused by SBI, however. In the current study, 14.83% (43/290) patients had bone metastasis and SBI was significantly associated with increased risk of bone metastasis (20.69% vs. 10.92% for SBI-positive vs. SBI-negative patients, respectively) and shorter BMFS [HR 2.43 (1.32–4.47), *P* < 0.05]. A Cox proportional hazard model with major covariable adjustment was used to examine the effect of SBI on bone metastasis. The results remained robust and stable even after three adjustments (*P* < 0.05). Yi et al. (14) demonstrated the predictive value of SBI for bone metastasis, particularly in patients with early N-staging NPC. While SBI was associated with poor DMFS and OS in this study, however, the covariable adjusted model showed that SBI may not be an independent factor. Feng et al. has demonstrated that extensive SBI is not an independent prognostic factor for DMFS and OS (28). In a separate study of 181 N0 NPC patients, the high-risk advanced T category, which included SBI, was an independent prognostic factor for PFS, OS, and locoregional relapse-free survival (29). Subgroup analysis assessed the relationship between SBI and bone metastasis based on TNM stage, IC, CCRT, and AC. Of note, no significant interaction effects were found in the four subgroups (*P* for interaction >0.05). There is a strong link between SBI and bone metastasis in different subgroups, which is consistent with prior studies (30–32). Collectively, these data confirm a correlation between SBI and a greater risk of bone metastasis.

This study suggests that the development of a prediction model of bone metastasis based on SBI is both feasible and meaningful. Collinearity, which exists in variables that are similar or have a strong association, should be checked before modeling, and variables with significant collinearity should not be included (33–35). Given that SBI and T category had strong collinearity (r = 0.657) in this study, while TNM stage did not (r = 0.293), TNM stage was selected for subsequent analyses. Chen et al. (36) developed a prognostic score for NPC patients with bone metastasis based on clinical routine factors. Another study (15) developed a nomogram using data from the Surveillance, Epidemiology, and End Results database to predict the prognosis of distant metastases. Yao et al. (37) used a nomogram to assess the benefits of adding IC to CCRT for N2-3 NPC patients and found that those in the high-risk group benefited more from combination therapy (DMFS: 69.5% vs. 56.7%, *P* = 0.004). There is no prediction model for bone metastasis risk in the Chinese population, however. Thus, a BMFS predictive model was developed based on SBI and visualized using a nomogram. The model included the following six components: SBI, KPS, TNM stage, IC, CCRT, and AC, and the nomogram performed well in both the training and validation datasets (C-index 0.80 vs. 0.79), which was consistent with the AUC at 2, 3, and 5 years. Calibration curves and DCA demonstrated the effectiveness of the nomogram. As a result, a nomogram based on SBI may provide an individual assessment of bone metastasis risk in LA-NPC patients.

The present study has several limitations. First, because it is a retrospective study, there is the possibility of both selection bias and confounding bias. Second, although the established nomogram model was trained and validated using data from two different hospitals, the sample size was small and external validation was not performed. Third, several variables including Epstein Barr Virus were not included in the analysis. Fourth, this study was conducted in an endemic area. So, extrapolation of the current results should be done with caution. Future studies should consider establishing an updated model with a large sample size and detailed data that is subject to external validation.

In conclusion, both unadjusted and adjusted analyses showed that SBI is strongly associated with the risk of bone metastasis. The established SBI-based nomogram can be used to assess the risk of bone metastasis in individual LA-NPC patients.

## Data Availability Statement

The raw data supporting the conclusions of this article will be made available by the authors, without undue reservation.

## Ethics Statement

The studies involving human participants were reviewed and approved by The Institutional Review Boards of Taizhou Central Hospital (Taizhou University Hospital). Written informed consent for participation was not required for this study in accordance with the national legislation and the institutional requirements.

## Author Contributions

BW and YT conceived the presented idea. YG and Q-GG collected the data. YG and HY analyzed the data, BW drafted the manuscript. YT improved the standard of English. All authors reviewed the manuscript and approved the submitted version.

## Funding

This study was funded by the Medical and Health Science and Technology Program of Zhejiang Province (2020RC040).

## Conflict of Interest

The authors declare that the research was conducted in the absence of any commercial or financial relationships that could be construed as a potential conflict of interest.

## Publisher’s Note

All claims expressed in this article are solely those of the authors and do not necessarily represent those of their affiliated organizations, or those of the publisher, the editors and the reviewers. Any product that may be evaluated in this article, or claim that may be made by its manufacturer, is not guaranteed or endorsed by the publisher.

## Supplementary Material

Supplementary Table 1 Baseline characteristics of 290 locally advanced nasopharyngeal carcinoma patients grouped by hospital.

Supplementary Table 2 Collinearity analysis of skull base invasion and covariables.
