# Supplementary material for: Predicting Bone Metastasis Risk Based on Skull Base Invasion in Locally Advanced Nasopharyngeal Carcinoma
Source: Front Oncol. 2022 Apr 7;12:812358. doi: 10.3389/fonc.2022.812358 (PMC9022773; doi:10.3389/fonc.2022.812358)
Supplement: Supplementary Table 1 — Baseline characteristics of 290 locally advanced nasopharyngeal carcinoma patients grouped by hospital. [file Table_1.docx]

**TABLE S1 |** Baseline characteristics of 290 locally advanced nasopharyngeal carcinoma patients grouped by hospital.

| **Variable** | **Total**  **(n = 290)** | **TZCH**  **(n = 198)** | **TZH**  **(n = 92)** | ***P* value** |
| --- | --- | --- | --- | --- |
| SBI |  |  |  | 0.938 |
| No | 174 (60.0) | 118 (59.6) | 56 (60.9) |  |
| Yes | 116 (40.0) | 80 (40.4) | 36 (39.1) |  |
| Bone metastasis |  |  |  | 0.685 |
| No | 247 (85.2) | 167 (84.3) | 80 (87.0) |  |
| Yes | 43 (14.8) | 31 (15.7) | 12 (13.0) |  |
| Age(years), Mean ± SD | 54.9 ± 11.6 | 54.7 ± 11.6 | 55.3 ± 11.6 | 0.686 |
| Age(years) |  |  |  | 1.000 |
| ≤55 | 146 (50.3) | 100 (50.5) | 46 (50.0) |  |
| >55 | 144 (49.7) | 98 (49.5) | 46 (50.0) |  |
| Sex |  |  |  | 0.767 |
| Female | 74 (25.5) | 49 (24.7) | 25 (27.2) |  |
| Male | 216 (74.5) | 149 (75.3) | 67 (72.8) |  |
| KPS scores |  |  |  | 0.866 |
| ≤70 | 66 (22.8) | 44 (22.2) | 22 (23.9) |  |
| >70 | 224 (77.2) | 154 (77.8) | 70 (76.1) |  |
| Smoking index |  |  |  | 0.376 |
| ≤400 | 207 (71.4) | 145 (73.2) | 62 (67.4) |  |
| >400 | 83 (28.6) | 53 (26.8) | 30 (32.6) |  |
| Histological type |  |  |  | 0.819 |
| Keratinizing | 22 (7.6) | 16 (8.1) | 6 (6.5) |  |
| Non-keratinizing | 268 (92.4) | 182 (91.9) | 86 (93.5) |  |
| T category |  |  |  | 0.546 |
| T1-2 | 114 (39.3) | 75 (37.9) | 39 (42.4) |  |
| T3-4 | 176 (60.7) | 123 (62.1) | 53 (57.6) |  |
| N category |  |  |  | 0.443 |
| N0-1 | 57 (19.7) | 36 (18.2) | 21 (22.8) |  |
| N2-3 | 233 (80.3) | 162 (81.8) | 71 (77.2) |  |
| TNM stage |  |  |  | 0.085 |
| III | 207 (71.4) | 148 (74.7) | 59 (64.1) |  |
| IVa | 83 (28.6) | 50 (25.3) | 33 (35.9) |  |
| IC |  |  |  | 0.421 |
| No | 125 (43.1) | 89 (44.9) | 36 (39.1) |  |
| Yes | 165 (56.9) | 109 (55.1) | 56 (60.9) |  |
| CCRT |  |  |  | 0.043 |
| No | 77 (26.6) | 45 (22.7) | 32 (34.8) |  |
| Yes | 213 (73.4) | 153 (77.3) | 60 (65.2) |  |
| AC |  |  |  | 1.000 |
| No | 183 (63.1) | 125 (63.1) | 58 (63.0) |  |
| Yes | 107 (36.9) | 73 (36.9) | 34 (37.0) |  |
| Abbreviations: TZCH, Taizhou Central Hospital (Taizhou University Hospital); TZH, Taizhou Hospital; SBI, skull base invasion; KPS, karnofsky performance status; IC, induction chemotherapy; CCRT, concurrent chemoradiotherapy; AC, adjuvant chemotherapy. | | | | |
