# Supplementary material for: Predicting Bone Metastasis Risk Based on Skull Base Invasion in Locally Advanced Nasopharyngeal Carcinoma
Source: Front Oncol. 2022 Apr 7;12:812358. doi: 10.3389/fonc.2022.812358 (PMC9022773; doi:10.3389/fonc.2022.812358)
Supplement: Supplementary Table 2 — Collinearity analysis of skull base invasion and covariables. [file Table_2.docx]

**TABLE S2 |** Collinearity analysis of skull base invasion and covariables.

| Code | SBI | Hospital | age | sex | KPS scores | Smoking index | Histological  type | T  category | N  category | TNM  stage | IC | CCRT |
| --- | --- | --- | --- | --- | --- | --- | --- | --- | --- | --- | --- | --- |
| Hospital | 0.012 | **-** | - | - | - | - | - | - | - | - | - | - |
| Age | -0.008 | -0.005 | - | - | - | - | - | - | - | - | - | - |
| Sex | 0.01 | 0.026 | 0.075 | - | - | - | - | - | - | - | - | - |
| KPS scores | 0.094 | -0.019 | 0.02 | 0.072 | - | - | - | - | - | - | - | - |
| Smoking index | -0.003 | -0.06 | 0.119* | 0.318** | 0.038 | - | - | - | - | - | - | - |
| Histological type | -0.032 | -0.027 | 0.076 | -0.078 | -0.093 | 0.124* | - | - | - | - | - | - |
| T category | 0.657** | 0.043 | -0.034 | -0.034 | 0.066 | -0.021 | -0.017 | - | - | - | - | - |
| N category | -0.181** | 0.054 | 0.04 | 0.029 | -0.042 | 0.044 | 0.022 | -.398** | - | - | - | - |
| TNM stage | 0.293** | -0.109 | -0.003 | 0.038 | 0.184** | 0.072 | 0.037 | 0.213** | 0.006 | - | - | - |
| IC | 0.213** | -0.055 | -.138* | 0.018 | -0.009 | 0.074 | 0.014 | 0.269** | -0.063 | 0.089 | - | - |
| CCRT | -0.019 | 0.127* | -.246** | -0.012 | -0.083 | -0.068 | -0.025 | 0.028 | -0.022 | -0.017 | -0.003 | - |
| AC | -0.026 | -0.001 | -.131* | 0.153** | 0.096 | 0.053 | -0.078 | -0.014 | -0.017 | -0.057 | -0.157** | 0.136* |
| Abbreviations: SBI, skull base invasion; KPS, karnofsky performance status; IC, induction chemotherapy; CCRT, concurrent chemoradiotherapy; AC, adjuvant chemotherapy.  ** P < 0.01, * P < 0.05 | | | | | | | | | | | | |
