# Supplementary material for: Predicting Bone Metastasis Risk Based on Skull Base Invasion in Locally Advanced Nasopharyngeal Carcinoma
Source: Front Oncol. 2022 Apr 7;12:812358. doi: 10.3389/fonc.2022.812358 (PMC9022773; doi:10.3389/fonc.2022.812358)
Supplement: Supplementary file 3 [file DataSheet_1.docx]

**Supplementary S1**

Chemotherapy:

- Induction chemotherapy

1. TPF: docetaxel 60 mg/m² iv, day 1 + cisplatin 60 mg/m² iv, day 1 + 5-FU 600 mg/m² iv, days 1–5 every 3 weeks
2. GP: gemcitabine 1000mg/m² iv, day 1 and 8 + cisplatin 25mg/m² iv, days 1–3 every 3 weeks
3. PF: cisplatin 80 mg/m² iv, day 1 + 5-FU 800 mg/m² iv, days 1–4/5 every 4 weeks

- Concurrent chemoradiotherapy

1. Cisplatin 30–40 mg/m² iv weekly
2. Cisplatin 80 mg/m² iv every 3 weeks

- Adjuvant chemotherapy

1. PF: cisplatin 80 mg/m² iv, day 1 + 5-FU 800 mg/m² iv, days 1–4/5 every 4 weeks
2. TPF: docetaxel 60 mg/m² iv, day 1 + cisplatin 60 mg/m² iv, day 1 + 5-FU 600 mg/m² iv, days 1–5 every 3 weeks
